# Supplementary material for: Are carbon emissions trading and green financial instruments synergistic? -Comprehensive quantitative research based on content analysis
Source: PLoS One. 2024 Mar 7;19(3):e0298601. doi: 10.1371/journal.pone.0298601 (PMC10919601; doi:10.1371/journal.pone.0298601)
Supplement: S1 Data — (ZIP) [file pone.0298601.s001.zip › Data/地级市Z_F工作报告.zip!/║■▒▒/╛ú├┼╩╨/└┤╫╘╣1⁄2╓┌║┼í╛┬φ┐╦╩2╛▌═°í┐_2012.docx]

各位代表：

　　现在，我代表市人民政府向大会作工作报告，请予审议，并请市政协各位委员和其他列席人员提出意见。

　　一、过去五年的工作回顾和2011年主要工作

　　五年来，在省委、省政府和市委的坚强领导下，市七届人民政府紧紧依靠全市人民，坚持以科学发展观为指导，扎实推进“四市”战略，积极应对国际金融危机等复杂多变的宏观环境和各种重大自然灾害，圆满完成了市七届人大一次会议确定的各项目标任务，谱写了荆门跨越发展的新篇章。

　　——这五年，我们致力优化经济结构、强化产业支撑，综合实力持续增强。全市经济进入加速发展期，主要经济指标实现翻番式增长，地区生产总值年均增长14.4%，规模工业增加值年均增长25.3%，财政总收入和地方一般预算收入年均分别增长32.5%、31.1%，全社会固定资产投资年均增长35.8%。三次产业比重由2006年的24.8：36.8：38.4调整为17.7：52.4：29.9。粮食总产实现“八连增”，国家级农业产业化龙头企业达到4家；工业拉动生产总值年均增长8.0个百分点；第三产业增加值年均增长13.0%。高新技术产业增加值年均增长19.0%。市场主体总户数达到8.8万户，非公有制经济比重由50.2%上升到54.6%。京山县、钟祥市、东宝区连年跻身全省县域经济20强，沙洋县在全省排位大幅前移。

　　——这五年，我们致力推进生态建设、完善城市功能，城乡面貌明显改观。全市城镇化率达到47%。中心城区建成区面积由2006年的44.6平方公里拓展到52平方公里，建成荆门生态运动公园、天鹅湖公园、李宁公园等一批标志性城市景观。3个县级城市、4个镇域小城市、各类集镇功能不断完善，新农村建设取得明显成效。生态荆门建设全面推进，提前两年完成“十一五”主要污染物减排任务，被确定为湖北省唯一的国家循环经济试点市，荣获国家现代林业示范市、全国绿化模范城市称号。交通、电力、通信、水利建设取得重大突破。

　　——这五年，我们致力深化改革开放、创新体制机制，发展活力不断释放。集体林权制度改革、医药卫生体制改革三年任务、政府机构改革圆满完成，文化体制及财税金融体制改革稳步推进。开放型经济加快发展，累计利用外资6.3亿美元，利用市外境内资金700亿元，引资项目2218个，中集、中海油、中粮、中储粮、华能、李宁、福耀、雨润、汇源、三一重工等“500强”企业和一大批知名品牌相继落户荆门。

　　——这五年，我们致力保障和改善民生、发展社会事业，和谐社会加速构建。城镇居民人均可支配收入和农民人均纯收入年均分别增长13.5%和14.2%。五大社会保险参保人数达到176.3万人次，基本医疗保险实现全覆盖，城镇职工养老保险参保人数快速增长，城乡居民社会养老保险制度不断完善，城乡低保实现应保尽保，困难群体帮扶救助体系进一步健全。住房保障体系加快建立。连续被评为全国科技进步先进城市。组建荆楚理工学院和荆门职教集团，义务教育各项指标高于全省平均水平。成功承办湖北省第十三届运动会和第八届中国艺术节分会场活动。社会和谐稳定，人民安居乐业。

　　各位代表，2011年是本届政府任期的最后一年，我们着力稳增长、调结构、惠民生，实现了“十二五”良好开局。全年实现地区生产总值942.59亿元，增长15.6%；规模工业增加值480.77亿元，增长26.2%；全社会固定资产投资590.63亿元，增长39.9%；社会消费品零售总额298.24亿元，增长18.3%；居民消费价格涨幅5.6%，超出控制目标0.7个百分点；完成财政总收入95.42亿元，增长19.4%，其中，地方一般预算收入39.39亿元，可比增长43.8%；实际外商直接投资额1.87亿美元，增长28%；出口3.83亿美元，增长28%；城镇居民人均可支配收入15526元，增长14.2%；农民人均纯收入8248元，增长18.7%；城镇登记失业率4.1%；人口自然增长率3.97％。；完成省定节能减排目标。除居民消费价格涨幅外，市七届人大五次会议确定的主要预期目标均可完成或超额完成。

　　一年来，我们主要做了以下八个方面的工作：

　　（一）抢抓“中国农谷”机遇，现代农业建设稳步推进。“中国农谷”建设被确定为省级发展战略重点，农业强市地位进一步彰显。着力克服三季连旱的影响，取得“一抗三保”重大胜利，粮食增产6620万斤，其他主要农产品产量稳步增长。全市农产品加工产值预计突破500亿元，增长29.5%，宝源木业、广源食品被评为国家级龙头企业。新增国家级农业标准化示范区1个、农产品地理标志产品2个、农业“三品”标志产品33个。彭墩、王坪、斗立、来龙、桥河等32个村被授予“全省新农村建设示范村”称号。58座小型水库整险加固、三干渠等灌区续建配套工程和大碑湾等泵站改造年度任务提前完工，扩挖、改造当家堰2.2万口。

　　完成土地整治26万亩，新增耕地面积1.85万亩。新建通村公路328.7公里、通组公路碎石路面1000公里、农村候车棚107个，解决10万人的农村饮水安全问题，发放丰产仓1.4万套。农机综合作业率达到68%。新增农民专业合作社166家，达到2321家，居全省首位。京山县、沙洋县、钟祥市“三农”发展综合实力考评进入全省10强。

　　（二）全面加快“一核六片十五园”建设，工业经济开始在同类城市中领跑。规模工业增加值总量全省名列第5位，增速全省第4位。新增规模以上企业62家，达到682家，新增销售收入过亿元的企业67家，达到255家。全市“1+6”园区完成基础设施建设投资33亿元，增长85%。荆门高新区、化工循环产业园体制机制进一步理顺，高新区完成工业总产值91.1亿元、税收4.3亿元，分别增长111.05%、231.3%。实现高新技术产业增加值7.5亿元，占规模工业增加值的比重达到14.5%；新增高新技术企业12家、省级工程技术中心和校企共建研发中心5家。成立“中国光谷·荆门产业园”，与中航集团共同打造航空新城，水泥行业重组壮大取得突破性进展。开展首届“市长质量奖”评选表彰活动，“宝源”获认中国驰名商标。

　　（三）着力在“鄂西生态文化旅游圈”中唱好“重头戏”，现代服务业的支撑作用逐步增强。京山绿林世纪广场、屈家岭月湖度假村、沙洋正中水镇等一批旅游项目建设扎实推进，彭墩村入选全国休闲农业示范点。先后举办油菜花旅游节、漳河游泳节、钟祥长寿文化旅游节等特色旅游活动，全年接待国内外游客1102.53万人次，实现旅游总收入53.07亿元，分别增长27.3%和31.0%。商贸物流业加快发展，东方百货新农村冷链物流、上海商贸城一期等项目投入运行，星球商业中心、宜洋汽车后市场等项目即将建成。交通银行进驻荆门，3家村镇银行相继组建营运。全市中小企业贷款余额达到182.4亿元，增长40.5%。

　　（四）扎实开展“中国光彩事业荆门行”活动，招商引资和项目建设取得新突破。成功举办6次光彩事业招商推介会和1次主体活动，共签约项目112个，投资总额530亿元。全市在建项目1907个，增长49.8%，其中新开工项目1707个。格林美稀贵金属资源化利用、航特科技工业园一期、广源食品工业园一期、伟嘉纺织工业园、巨星粮油深加工等47个亿元项目竣工投产，荆门石化扩能改造、天茂丙烯工程、龙池粮油产业园、荆新一级公路、旅游大环线等150个亿元项目顺利推进或基本建成，大连实德科技综合产业园等30多个重大项目前期取得实质性进展。

　　（五）全力推进“国家园林城市”及“楚天杯”创建工作，城市规划建设管理水平有了新提高。顺应城市发展需要，成立了漳河新区。启动了新一轮城市总体规划修改，编制了中心城区七大湖水系保护、漳河“三圈”保护线、道路命名、城市综合交通等专项规划和CBD概念规划，开展了市政务中心片区、高速公路出入口的城市设计，完成了土地利用总体规划修编工作。投资7.2亿元开工建设40个城建项目，长坂坡路、杨湾桥新桥、东宝山隧道改造等工程竣工通车，漳河大道、三水厂管道取水工程按计划推进。实行城市规划建设管理和重大项目“5+2”周六例会制度，成立城市管理委员会，设立区级城管局（城管执法局），积极开展控违拆违和环境卫生、户外广告等专项整治，大力实施园林绿化工程，创建国家园林城市以优异的成绩通过国家住建部综合评审，争创“楚天杯”以全省总分第三名的成绩通过综合考评。新投放公交车60辆，公交线路进一步优化。镇域小城市建设进程加快，机构编制调整和首批下放事权工作全面完成。

　　（六）深入开展“国家循环经济试点市”建设，环境保护和节能减排工作成效明显。实施循环经济项目145个，总投资125亿元。荆门化工、格林美、荆襄磷化三大循环产业园发展步入快车道，我市被授予国家循环经济教育示范基地。实施节能产品生产及技改项目52个、减排项目24个。荆门高新区污水提升泵站和杨树港污水处理厂投入使用。葛洲坝水泥厂异地技改新址基本确定。县级以上饮用水源达标率100%，中心城区空气质量达标率高于国家标准，竹皮河水质多项指标恢复四类标准。

　　（七）认真落实“政府10件实事”，各项社会事业协调发展。持续开展“千名干部进千村入万户”和“万名干部进社区”活动，解决了一批事关城乡群众切身利益的突出问题。城镇新增就业4.6万人，扶持创业5160人，农村劳动力转移就业5.2万人。实现医疗保险市级统筹和基本药物制度基层全覆盖，城镇居民社会养老保险启动实施，新型农村社会养老保险试点稳步推进。启动物价上涨与社会救助、保障标准挂钩联动机制，农村低保新增1.25万人。实施医疗救助和临时救助3.3万人次，发放救助资金2186万元。开工建设各类保障性住房16931套，其中改造各类棚户区10406户，发放廉租住房租赁补贴2332万元。新建、改建农村乡镇中心幼儿园30所；维修改造中小学校舍2.9万平方米，为1.6万名义务教育阶段困难寄宿生提供生活补助，资助1.1万名家庭经济困难中职生完成学业，对市特殊教育学校在校学生实行“四免”政策。全面消除基层医疗卫生机构危房和卫生室服务空白村。建设村级文化活动中心60个、农民健身工程210个，农家书屋实现全覆盖。办理各类法律援助案件910件。组建荆门日报传媒集团，完成广电网络整合和市艺术剧院改制主要工作。我市被授予“全国人口和计划生育综合改革示范市”称号。城乡建设用地增减挂钩模式得到国土资源部肯定。国防教育、“双拥”、民防和民兵预备役建设不断加强。地方志工作步入全国先进行列。统计、物价、工商、民族宗教、扶贫开发、移民、血防、烟草、外事侨务、应急管理、机关事务管理、气象、档案、保密和工会、青年、妇女、儿童、对台、老龄、残联、无线电、住房公积金管理等各项工作都取得新成绩。

　　（八）大力整治“庸、懒、散”，政府自身建设切实加强。共办理人大议案建议111件、政协提案326件。圆满完成第七届村委会和第三届居委会换届选举。“六五”普法全面启动。信访非访总量持续下降，安全生产形势平稳，人民调解成功率达95.8%。积极推行复式执法，严厉打击违法犯罪，人民群众的安全感不断提高。实施行政审批制度改革，市本级行政审批事项从365项减到190项，为全省最少；服务时限提速25%。京山县行政审批零收费试点工作成效明显，市直零收费改革方案开始实施。深入开展“解放思想、跨越发展”主题实践活动和整治“庸、懒、散”活动，全市公务员队伍履职尽责意识明显增强。加大对重点部门、重点领域的审计监督，严肃财经纪律，审计创新发展连续两年获得全省考核第一名。强化行政、执法、廉政和效能监察，深化源头治腐各项改革，扎实开展以工程领域突出问题为重点的专项治理，狠抓招投标综合监管体系建设，廉政建设进一步加强。

　　各位代表，成绩来之不易，奋斗充满艰辛。这些成绩的取得，归功于省委、省政府和市委的正确领导，归功于市人大、市政协和社会各界的监督支持，归功于历届政府班子的励精图治，更归功于全市人民的团结拼搏。在此，我代表市人民政府，向全市广大干部群众，向各民主党派、工商联、无党派人士和人民团体，向驻荆部队、武警官兵和公安干警，向关心、支持、帮助荆门发展的社会各界朋友，表示衷心的感谢并致以崇高的敬意！

　　回顾过去五年的实践，我们深刻体会到，做好政府工作，必须把解放思想、科学发展作为最鲜明的发展导向，不断在应对挑战中转变观念，在做大经济总量中推进结构调整；必须把深化改革、扩大开放作为最强劲的发展动力，坚定不移地推进投资拉动，狠抓项目、园区建设和体制机制创新；必须把节约资源、保护环境作为最紧要的发展要求，积极发展循环、低碳、生态经济；必须把以人为本、改善民生作为最根本的发展目的，不断满足全市人民过上幸福生活的新期待；必须把勤政务实、廉洁高效作为最有力的发展保障，敢于担当，多谋善断，勇于创新，狠抓落实。

　　在总结成绩和经验的同时，我们也清醒地看到前进中还存在不少问题。主要是：经济规模仍然偏小，重大项目储备不多，结构调整任务较重，科技创新能力不强，经济发展的质量和效益有待进一步提高；土地供应趋紧与集约利用率不高、“就业难”与“用工难”、资金需求与供给等矛盾并存；城市与工业污染物排放基数较高、治理难度较大，农村面源污染、生活污染还较为严重，工程性减排措施有待进一步加强；城乡规划比较滞后，城镇建设水平有待提高，城市管理中存在的问题还比较突出；城乡居民收入稳定增长的渠道不够宽，社会保障水平仍然较低；城乡优质教育、医疗资源不足，分布不均；一些部门办事效率不高，少数政府工作人员危机意识不强，缺乏工作激情；此外，极少数公务员的群众观念比较淡薄，腐败问题还时有发生，等等。对此，我们将高度重视，认真对待，下大力解决。

　　二、新一届政府的总体要求和目标任务

　　今后五年，是荆门推进“三化同步”、建设“中国农谷”、实现跨越崛起的关键时期，也是国际国内竞争加剧、资源环境约束趋紧、社会建设和管理任务加重的矛盾攻坚期，又是加快转变经济发展方式、提升综合竞争力的发展机遇期。按照市第七次党代会的战略部署，新一届政府工作的总体要求是：以邓小平理论和“三个代表”重要思想为指导，深入贯彻落实科学发展观，高举“中国农谷”旗帜，紧扣全面建成小康社会和综合实力走在全省同类城市前列的“两大目标”，强力推进新型工业化、新型城镇化和农业现代化“三化同步”发展，加快建设实力、文化、生态、幸福“四个荆门”，奋力实现跨越崛起，成为湖北构建“战略支点”的重要支撑。

　　目标任务是：

　　——全力打造实力荆门。主要经济指标在全省位次前移，地区生产总值、工业增加值、财政总收入实现翻番，分别达到2000亿元、1000亿元、200亿元以上。高新技术产业增加值占规模工业增加值的比重提高到18%。城镇化率达到65%，城镇规划建设管理水平进一步提高。

　　——全力打造文化荆门。市民文明素质显著提高，公共文化服务体系基本形成，培育10家以上过亿元的文化产业示范企业，文化产业增加值占生产总值的比重达到6%左右，文化软实力进一步增强。

　　——全力打造生态荆门。单位生产总值综合能耗比2011年下降16%，化学需氧量、二氧化硫、氨氮、氮氧化物排放分别比2011年削减7.4%、8.8%、10%和7.2%，森林覆盖率达到40%。

　　——全力打造幸福荆门。城镇居民人均可支配收入、农民人均纯收入分别超过3万元和1.5万元，中等收入群体持续扩大。覆盖城乡的社会保障体系更加健全，基本公共服务水平和均等化程度大幅提高。

　　工作重点是：

　　（一）抓产业、抓环境。强化产业强市理念，全地域、全产业、全体系推进“中国农谷”建设，初步建成“四地一区一市”。实施工业“千百十”工程，着力培植化工、农产品加工两大千亿产业和荆门高新区、化工循环产业园两大千亿园区，倾力打造10家左右百亿企业，发展一批行业领军企业，力争销售收入过10亿元的企业超过100家。加快建设国家现代农业示范区，建成全国有重要影响的农产品生产加工聚集区、农业生态旅游区、农耕文化展示区、农业科技示范区，力争农业现代化走在全省前列。大力发展以生态文化旅游、生产性物流为重点的现代服务业，努力实现服务业增加值增速高于经济总量增速。实施开放先导战略，切实形成全方位、多层次、宽领域的开放格局。深入实施行政审批制度改革，认真抓好财政、土地、投融资和公务员管理体制改革，把荆门打造成全省审批项目最少、流程最短、成本最低、效率最高的城市。建立城乡环境综合治理长效机制，实施城市管理“三年行动计划”，积极创建国家卫生城市和环境模范城市，努力使荆门天更蓝、地更绿、水更清、景更美。

　　（二）建园区、建新城。坚持产业集群、要素集中、生态集约、管理集成的原则，加快推进“一核六片十五园”建设，力争到2016年，全市“1+6”园区完成总产值5000亿元。按照中心城区“四大功能区”统筹协调发展的思路，扎实抓好西片市级政务中心和“零工业污染”生态宜居新区、南片国家级高新技术产业园区、东片化工循环产业园区、北片东宝都市工业园区建设，建成“三环八射”、进出方便的城市道路体系，打造鄂中区域性大城市；鼓励和扶持县级中等城市、镇域小城市、工业重镇、旅游名镇竞相发展，力促鄂中城市群加快崛起。积极推进交通等重大基础设施建设，形成“一小时经济圈”，增强荆门“同城效应”。

　　（三）重人才、重效率。跨越亟需提高效率，崛起更需人才支撑。完善人才政策体系，深入推进龙泉英才计划、象山学者计划、金农能人计划，大力实施“金蓝领”工程，培养引进领军型人才、创新创业团队、优秀企业家，以及金融服务、资本运作、创意产业等高端人才，全面提升人才竞争优势。健全面向基层、面向群众、面向企业和高效快捷、运转顺畅的工作机制，完善信息化工作平台，切实提高政府执行力和办事效率。

　　（四）促民生、促和谐。进一步拓宽增收渠道，努力实现“城乡居民收入倍增”目标。注重完善保障体系，确保养老保险、医疗保险、社会救助、住房保障实现“全覆盖”。着力扩大公共服务，更好地解决人民群众在就业、就医、就学、养老、住房等方面存在的问题。扎实推进平安创建，深入开展“六五”普法，狠抓安全生产，切实维护社会稳定。坚持每年为群众办几件看得见、得实惠的好事实事，不断提高发展的包容性，使全市人民的生活过得更加殷实、更加安康、更加幸福！

　　三、2012年的政府工作

　　2012年是新一届政府的开局之年，是实施“十二五”规划承上启下的重要一年，做好政府各项工作，具有十分重要的意义。要积极应对经济增长下行和物价上涨双重压力、部分企业生产经营困难、节能减排形势严峻、要素“瓶颈”制约突出等矛盾和问题，抢抓省委、省政府支持我市建设“中国农谷”的大好机遇，充分发挥我市实体经济基础比较扎实的优势，坚持稳中求进、好中求快、又好又快，全力推进经济社会跨越式发展，努力实现“千亿经济总量、百亿财政收入”目标。全年主要预期目标是：地区生产总值增长13%以上，跨入全省“千亿俱乐部”；规模以上工业增加值增长22%，保持在同类城市前列位置；全社会固定资产投资增长25%；社会消费品零售总额增长18%；居民消费价格涨幅控制在4.5%左右；财政总收入增长12.6%，地方一般预算收入增长17%；出口总额增长16%，实际外商直接投资增长12%；城镇居民人均可支配收入、农民人均纯收入分别增长12%；城镇登记失业率控制在4.3%以内；人口自然增长率稳定在4.5％。以内；单位生产总值综合能耗、主要污染物排放量削减率完成省定目标。

　　今年要突出抓好以下工作：

　　（一）推动经济平稳较快发展。

　　全面推进“中国农谷”建设，促进现代农业高端发展。围绕建设国家现代农业示范区目标，高标准编制“中国农谷”总体规划及各类专项规划。强化“中国农谷”产业支撑，加强粮棉油等优质农产品生产基地建设，推广高效渔业养殖模式，提高畜禽标准化养殖场覆盖面，扩大蔬菜种植面积；提升京山、沙洋、钟祥农产品加工园区和东宝森工科技产业园的规划建设水平，力争新增年销售收入过10亿元的龙头企业4家，过30亿元的1家。创新“中国农谷”建设体制机制，抓紧组建屈家岭“中国农谷”省级开发区管委会和荆门农谷产业发展、建设、投资平台；设立“中国农谷”发展基金，引进各类市场主体，拓宽融资渠道；积极发展农村土地存贷合作社和流转信息中心，推进土地适度规模经营；加大小型农田水利产权改革力度；巩固和完善农村公益性服务“以钱养事”机制。规范农村集体资金、资产、资源管理，壮大集体经济，化解村级债务。坚持规范运作，提高农民专业合作社服务能力。健全农业技术推广、动植物疫情防控和农产品质量安全监管体系，力争农机综合作业率达到69%以上，新认证农业“三品”30个以上。加强农田水利基本建设，完成65公里汉江堤防崩岸整治、100座小型水库除险加固和4条中小河流整治任务，抓好4个小农水重点县建设，疏通沟渠5000公里。实施低丘岗地改造2万亩、土地整治20万亩。

　　深化“一核六片十五园”建设，促进工业经济提速增效。加快推进“1+6”园区配套基础设施建设，创新管理体制和服务方式，提高产业承载力，力争全市各类园区总产值达到1000亿元以上；荆门高新区实现技工贸总收入200亿元，税收6亿元。坚持项目带动，促进骨干企业进一步做大做强，重点开工建设华能热电联产、实德科技综合产业园、绿辰华顿甲醇汽（柴）油、湖北财富农业科技园、海鸥卫浴工业园、德力西电器设备制造等项目，扎实推进中航荆门航空新城、李宁产业园加工园区、格林美城市矿产资源循环产业园、洋丰中磷二期、玉皇聚苯乙烯、屈家岭安能生物质发电等项目，确保天茂丙烯工程、中集能源装备二期、金龙泉米糠蛋白一期等项目建成投产。建立健全地方政府与中省企业“双向纳入”机制，支持荆门石化实施“116”工程，抓紧启动1000万吨扩能改造项目。实施中小企业成长工程，力争新增规模企业80家，新增年销售收入过10亿元的企业20家。加快新洋丰、宝源木业等企业上市的培训和辅导步伐，力争凯龙IPO顺利过会。提高银政企合作效率，确保信贷投放增速高于全省平均水平、高于全市经济增长水平，中小企业贷款增速高于全市贷款增速。

　　大力发展现代服务业，促进第三产业扩量升级。编制并实施明显陵-漳河、大洪山、纪山楚文化三大旅游区总体规划和控制性详细规划。抓好京山温泉主题公园、沙洋农业四季花开生态风情园、大洪山陈湾温泉、马河青林寨红叶古堡景区、屈家岭美好人间、太子山国际狩猎场等重点旅游项目建设，做好屈家岭文化遗址公园等项目前期工作。启动绿林山风景区创建国家“5A”级景区工作，支持漳河风景区、彭墩乡村旅游度假区创建国家“4A”级景区，办好油菜花旅游节、京山温泉网球旅游节等节会，力争全市全年接待国内外游客达到1350万人次，旅游总收入达到65亿元。大力发展现代物流业，加快推进李宁物流园、众诚批发及冷链物流、多辉农产品交易中心等项目建设，切实做好蒙西至华东地区铁路煤运通道、荆门港石牌核心港区、荆门公铁物流中心等项目前期工作。积极发展总部经济，大力推进国华人寿、天平保险全国电销中心等项目建设。加快发展现代金融业，力争全市3家农村信用联社改制为农村商业银行，引进1-2家股份制商业银行在我市设立分支机构，组建1-2家村镇银行、5-6家小额贷款公司。设立中小企业金融服务网，改善小微企业和“三农”金融服务。推进政策性保险试点，整顿和规范保险业经营秩序。

　　（二）加快转变经济发展方式。

　　努力扩大内需。新建或续建230个亿元以上项目，完成投资380亿元；调度50个重大项目，完成投资120亿元。引导农产品流通企业建立直采基地，促进重点城镇综合商业中心及“销品茂”等新型业态发展。启动工人文化宫城市商业综合体、掇刀汉正街综合改造等项目，支持东方、中商、金城等商贸企业加快发展。引导发展信贷消费、租赁消费和网络消费，促进居民文化、旅游、健身、养老、家政等服务消费。千方百计保障市场供应，努力保持物价基本稳定。坚决打击商业欺诈、制假售假行为，让广大群众放心消费、安全消费。

　　加快自主创新步伐。继续开展“院士专家荆门行”、“厅市合作”等活动，鼓励大中型企业与高校、科研院所联建研发中心、中试和产业化基地，支持格林美申报国家城市矿产资源工程技术研究中心。大力发展环境修复、先进制造、新材料、电子信息、节能环保与新能源等战略性新兴产业，新申报认定高新技术企业15家以上。坚持以创促建，力争荆门高新区跃居国家高新区行列。成立荆门农业科学院，创办“中国农谷”论坛，引进国内外知名农业科研院所在我市设立科研、试验基地。实施“质量兴市”、“品牌兴市”战略，保护、培育一批精品名牌。

　　大力发展开放型经济。健全招商引资目标责任制和招商奖励、招商代理、投资协调等制度，以产业为主线，以园区为载体，有针对性地大招商、招大商，切实提高引进项目的投资强度、科技含量和产业带动水平。力争实现外商直接投资1.89亿美元、引进市外境内资金260亿元、出口4.32亿美元以上。鼓励扶持优势企业“走出去”布点经营，扩大对外劳务合作，提高外经合作水平。大力推进多层次资本市场建设，积极发展担保业和BOT、BT、PE、VC等投融资模式，支持发行企业债券和中期票据，力争城投债券早日发行。加强与国内外友好城市及长江中游城市群等地区的经济社会协作，促进共同发展。

　　提升循环经济发展水平。深入推进三大循环产业园建设，组织实施重点循环经济项目120个，完成投资100亿元。积极开展农业废弃物综合利用，大力推广新型种养循环模式，继续推进沼气、太阳能等农村清洁能源工程建设。推广使用新型环保建筑材料，建设可再生能源建筑应用国家示范城市。支持格林美探索建立废旧家电回收的社会商业模式，争创国家城市矿产资源产业示范基地和再生资源回收体系试点市。

　　加大节能减排和环境保护力度。认真执行环评审批和项目能评审查制度，创新节能管理机制，加强公共机构节能工作。坚持用最严格的措施保护漳河，加大长湖污染治理力度,严肃查处水库投肥养殖行为，整治取缔威胁水源安全的污染隐患。深入开展竹皮河流域污染综合治理工作，力争葛洲坝水泥厂技改搬迁取得实质性进展。严格危化企业环境管理。加强矿山地质环境的治理恢复，做好地质灾害隐患区点的排查和监测预警。加快乡镇污水、垃圾处理设施建设步伐。着力防治畜禽集中养殖污染，有效降低农村面源污染。创新碳汇造林机制，持续推进林业产业发展，力争全市完成人工造林30万亩。

　　（三）全力推进鄂中城市群建设。

　　狠抓各类规划修编。完成新一轮城市总体规划修改和城中村综合改造规划编制工作。高起点、高标准、高水平编制漳河新区总体规划和漳河“三圈”、荆门高新区等重点区域控制性详细规划，编制中心城区给排水、消防、电力、通信、环卫、社会停车场等专项规划。完善县城、镇、村规划，进一步扩大控制性详细规划的覆盖面。严格规划管理，切实维护规划的严肃性和权威性。

　　加快中心城区建设。大手笔、大气魄推进漳河新区建设，加快西外环、漳河大道、双喜大道等主干道建设步伐，启动建设市政务中心、CBD等项目，突破性推进荆门火车站工程、公铁换乘中心、“五湖连通”等项目前期工作。新建掇刀石大道、清水源路等南城区道路，全面开展象山大道、白云大道、虎牙关大道和兴隆大道的综合整治。做好已关闭采石场的生态复绿工作，加快象山风景区、东宝山·千佛洞风景区和市植物园建设，新建凤凰湖、双泉、杨湾公园和银河游园等城市绿化精品。建立荆门市地理信息中心，加快“数字荆门”建设。组建荆门城建集团。

　　切实加强城市管理。全面实施新的城管体制机制，构建数字化城市管理监督和指挥体系，实行网络化管理，着力解决“脏、乱、差”等突出问题。改革城市生活垃圾处理费征收方式，提高征收率。积极推进城市“慢行系统”建设，实施城区停车收费，逐步缓解城市交通压力。按照规范、整洁、便民的标准化要求，规划改造象山、南台等菜市场。坚决打好“拆违控违”攻坚战，确保中心城区违法建设零增加，违建防控率、顶风违建拆除率均达100%。

　　深入推进城乡一体化。促进县域经济持续快速发展，努力实现“保强进位”目标。完善市区分税制财政管理体制，增强区级发展能力。推动县级城市建管再上新水平，支持京山县争创全省生态文明第一县、沙洋县建设汉江流域“明珠城市”、钟祥市向现代园林型中等城市迈进。以镇域小城市为重点，大力推进中心镇建设。以“一村五镇三区”为试点示范，由点及面、整体联动，提高新农村建设水平。加快建设武荆高速公路屈家岭连接线（农谷大道）、京山连接线、沙洋连接线，完成国省道大中修100公里，改扩建二级公路100公里。实施新一轮农村电网改造升级工程，完善农村饮水安全工程设施，提高入户率；加强农村气象灾害防御能力建设，不断改善农村生产生活条件。

　　（四）促进文化大发展大繁荣。

　　培育城市文明风尚。大力实施市民文明素质提升工程，深入开展群众性精神文明创建活动，扎实推进社会主义核心价值体系建设。积极开展市民文明行为主题实践活动，巩固国家园林城市和“楚天杯”创建成果。抓好村镇、社区、校园、企业、家庭文化建设。加强人文关怀，注重心理疏导，培育奋发进取、理性平和、开放包容的社会心态。

　　健全公共文化服务体系。启动市博物馆、青少年及妇女儿童活动中心、荆楚文化广场（文化综合体）规划建设，建立社区文化长效机制，完善乡镇文化站和农村公共文化服务设施。扩大数字电视覆盖面，广泛开展“健康荆门”系列全民健身运动。加强优秀文化产品创作生产，推出一批在全省、全国有影响的精品力作。加强文物保护和利用。完成公益性文化事业单位改制任务，转变农村文化管办方式。策划并办好中国国际航空体育旅游节和荆门市第六届运动会。

　　提升文化产业整体实力。着力发展新闻传媒、演艺娱乐、体育用品、体育休闲和健身、出版发行等重点产业，支持荆门日报传媒集团做大做强，组建荆门广播电影电视传媒集团、荆门文化体育产业集团，加快发展影视制作、动漫等新型文化业态。编制和包装一批文化项目，大力开展文化招商活动，逐步发展壮大文化产业。

　　（五）着力建设“幸福荆门”。

　　扎实做好就业和创业工作。深入推进国家级创业型城市创建，支持劳动密集型产业和小型、微型企业发展，多渠道开发就业岗位。抓好“充分就业社区”建设，加强农村劳动力就业技能培训，积极帮助新生代农民工、返乡农民工、高校毕业生、就业困难人员实现就业，努力实现农村劳动力转移就业5万人，帮助下岗失业人员再就业1万人。

　　进一步完善社会保障体系。加大社会保险扩面征缴力度，力争五大社会保险参保人数达到177万人次，巩固城乡医疗保险，实现城乡居民养老保险全覆盖。进一步完善社会救助体系，推进城乡低保动态管理、应保尽保，实现困难群众临时救助、医疗救助、法律服务全覆盖。规范征收价格调节基金，对低收入群体给予动态价格补贴。启动荆门市失能半失能老人托养中心、“千福园”老年公寓二期建设工程，加快发展养老服务业。

　　大力发展各项社会事业。继续开展科普示范县（市、区）创建，有效提升全民科学素质。全面实施中小学标准化建设和农村中小学教师周转房建设试点、教育信息化工程，加快中小学布局调整步伐，促进义务教育公平和优质均衡发展。启动南城区3所新校建设。继续实施学前教育“三年行动计划”，构建广覆盖、保基本、有质量的学前教育公共服务体系。促进高级技工学校加快发展，规划建设荆门职教产业园区，启动高职院校筹建工作。支持荆楚理工学院建设和发展，使其与地方经济社会发展高度融合。强化校车及学生校外托管中心管理，加强学校及周边环境综合治理。巩固医疗卫生体制改革成果，完善医保市级统筹运行机制，稳步开展医疗保险异地就医前台结算；以城乡居民健康档案电子化为重点，全面实施基本公共卫生服务；加快南城区医院建设步伐；进一步加强血防工作。综合治理出生人口性别比，稳定低生育水平。全面落实移民后期扶持政策，继续做好对口援疆（精河县）、援恩（巴东县）工作。深入实施妇女儿童发展规划，依法保障妇女、未成年人和残疾人的合法权益，高度重视老龄事业发展及关心下一代工作，提升荆门市老年大学建设水平。深入开展双拥共建活动，认真落实军人及军属优抚安置政策，做好军队转业干部、复员退伍军人接收安置工作。进一步完善住房公积金制度，深入推进扶贫开发，加强统计“四大工程”建设，做好通信、邮政、烟草、盐业、成品油、天然气、无线电工作，积极推进档案、民防、地方志、外事侨务、民族宗教、台湾事务、仲裁、气象等社会事业发展。

　　加强社会管理创新。建立健全源头治理、动态协调和应急处置相互衔接、相互支持的机制，从源头上、根本上解决问题。全面推进城市网格化管理，提高城乡社区自治和服务功能。总结推广京山县“湾长理事”、沙洋县“法治高阳”、掇刀区“和事老工作室”经验，及时化解基层矛盾。坚持用群众工作统揽信访工作，全面落实维稳工作责任制，进一步完善社会稳定风险评估机制。加强流动人口、特殊人群、农村留守家庭、社会治安重点地区以及社会组织的服务管理，抓好虚拟社会动态管理和网络舆情引导。严格落实安全生产责任制，强化矿山、化工、建筑以及特种设备等重点行业的安全监管，积极开展全省食品药品安全先进市创建活动。加强社会治安综合治理，依法严厉打击各种犯罪活动，扎实推进“平安荆门”建设。

　　各位代表，悠悠万事，民生为大！今年，新一届人民政府将继续为人民群众办好10件实事：

　　1.完成三水厂管道取水工程建设，确保当年投入使用。

　　2.全市城镇新增就业4.4万人，通过政策扶持创业5000人、带动就业2万人。

　　3.建设和完善覆盖城乡的四级行政便民服务网络体系；为农民提供丰产仓1万套；提档升级村级服务社300家。

　　4.完善中心城区基础设施建设，新建10个社区服务中心，改造背街小巷30条，新建10条街巷路灯，建设金柏润公共人防地下工程。

　　5.按每生3000元标准资助100名应届大学新生入学，定向资助500名困难农民工子女上学，新建或改扩建幼儿园30所。

　　6.启动广播电视发射塔迁建工作，并将原址改建为绿化小游园，丰富千佛洞国家森林公园景观。

　　7.新增廉租住房租赁补贴2000户，新建廉租住房868套、经济适用住房1600套、公共租赁住房3500套、限价商品房500套，改造各类棚户区7868户。

　　8.对翠竹园、果园、蔡湾、十里牌等4家菜市场进行标准化改建，完善苏畈桥、东山、千汇、通源等4家菜市场配套设施。

　　9.新建通村公路200公里，改造县乡道100公里。扩挖、改造当家堰1万口。建设40个村级文化活动中心、150个农民体育健身工程。

　　10.为困难群众及特殊群体免费提供法律援助。

　　四、加强政府自身建设

　　各位代表，“意莫高于爱民，行莫厚于乐民。”我们将以建设责任政府、法治政府、效能政府、廉洁政府为重点，倾力打造对人民负责、为人民办事、让人民满意的“民本政府”。

　　（一）坚持强化责任，在拼搏实干上有更大进步。广泛开展爱岗敬业教育，增强政府工作人员的岗位责任意识。加强部门团结协作，建立健全部门主办责任制、联席会议制，坚决克服各行其是、推诿扯皮的行为，确保政令畅通。不断完善政府全年重点工作督查、阶段性工作推进和基层问题“现场办”机制，对确定的目标任务，要抢先机、快推进；在难题面前，要敢闯敢试、敢为人先；在矛盾面前，要敢抓敢管、敢于碰硬；在风险面前，要善于化解、敢于担当，真正做到履职有责、失职必究、执行有力、运转高效。

　　（二）坚持健全机制，在依法行政上有更高标准。自觉接受市委领导和人大及其常委会的法律监督、工作监督以及政协的民主监督，严格执行人大及其常委会的决议、决定，广泛听取政协及各民主党派、工商联、无党派人士的意见和建议，认真办理人大议案、代表建议和政协委员提案。深化行政执法体制改革，完善推进依法行政的工作制度和考核办法，加强行政复议能力建设，落实行政首长出庭应诉各项规定。完善政府公开征求意见的工作机制，最大限度推进政务公开透明，让人民群众充分知情、理解、参与和监督。

　　（三）坚持提高效能，在转变职能上有更实举措。设置专业技术官职位，推进公务员流动管理，清理、减少和调整行政审批事项，大力推行行政审批零收费。深化电子政务建设，推进电子化招投标，形成社会管理、公共服务事项网上一口受理、在线协同办理机制。牢固树立“有危机、快几拍”的思想，继续推行“三少三短”新政，少开会、开短会，少讲话、讲短话，少发文、发短文，把更多的精力和时间用在为群众、为基层办实事上，用在服务企业、推进项目建设上。

　　（四）坚持从严治政，在廉政勤政上有更好形象。更加注重治本、注重预防、注重制度建设，把廉政的要求落实到每个工作岗位上，落实到每项权力行使过程中。加强对中央和省委、市委各项重大决策部署贯彻执行情况的监督检查，强化对重点工作、重大项目的审计监督，深化领导干部经济责任审计，狠抓建设工程、土地出让、产权交易、医药购销、政府采购等领域的招投标综合监管。规范地方政府债务和预算绩效管理，严格控制“三公”经费支出，稳步推进财政预算信息公开，深化机关事务管理改革，降低行政成本。深入推进专项治理工作，坚决纠正损害群众利益的不正之风，严肃查处各种违法违纪案件。深入开展“庸、懒、散”整治活动，切实加强公务员队伍建设，不断提高人民群众对政府的满意度和信任度。

　　各位代表，美好的蓝图已经绘就，发展的责任重于泰山，人民的利益至高无上。让我们紧密团结在以胡锦涛同志为总书记的党中央周围，在中共荆门市委的坚强领导下，紧紧依靠全市人民，齐心协力、开拓创新，为全面实现市第七次党代会确定的宏伟目标而努力奋斗！
